# Supplementary material for: Clinical and economic outcomes of adding [18F]FES PET/CT in estrogen receptor status identification in metastatic and recurrent breast cancer in the US
Source: PLoS One. 2024 May 14;19(5):e0302486. doi: 10.1371/journal.pone.0302486 (PMC11093585; doi:10.1371/journal.pone.0302486)
Supplement: S6 Fig — (DOCX) [file pone.0302486.s006.docx]

**Supporting Information**

**Fig. S6 Tornado Diagram: OWSA for the 10 most sensitive parameters** in rBC patients when [18F]FES PET/CT is used in combination with IHC.


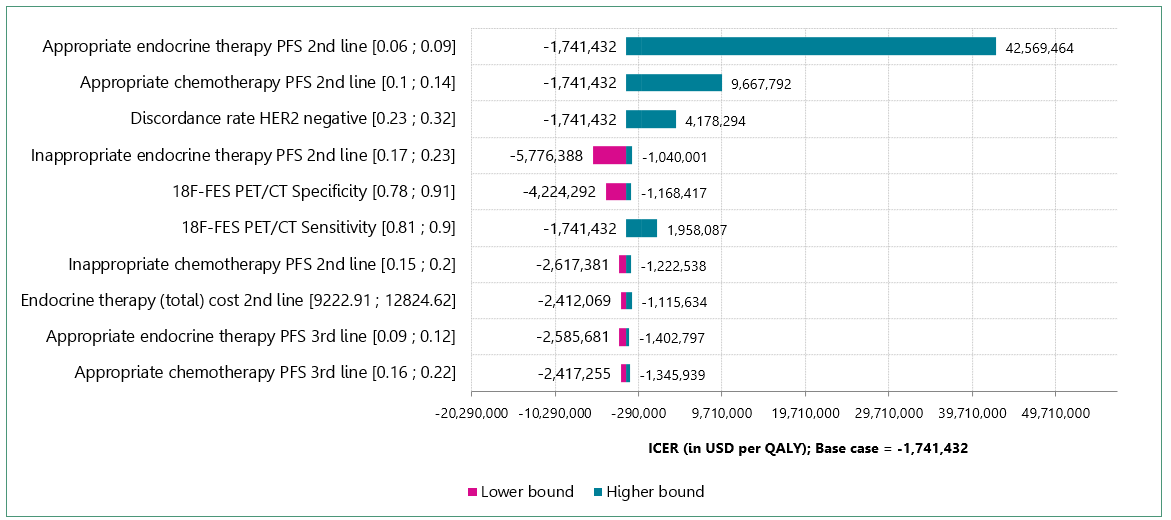


[^18^F]FES PET/CT indicates 16α-[^18^F]fluoro-17β-fluoroestradiol with positron emission tomography imaging/computed tomography; IHC, immunohistochemistry; HER2, human epidermal growth factor receptor 2; PFS, progression-free survival; mBC, metastatic breast cancer; OWSA, one-way sensitivity analysis; rBC, recurrent breast cancer.
