# Supplementary material for: Clinical and economic outcomes of adding [18F]FES PET/CT in estrogen receptor status identification in metastatic and recurrent breast cancer in the US
Source: PLoS One. 2024 May 14;19(5):e0302486. doi: 10.1371/journal.pone.0302486 (PMC11093585; doi:10.1371/journal.pone.0302486)
Supplement: S8 Fig — (DOCX) [file pone.0302486.s008.docx]

**Supporting Information**

| **Fig. S8 Cost-effectiveness plane** for mBC patients when biopsy was not possible.  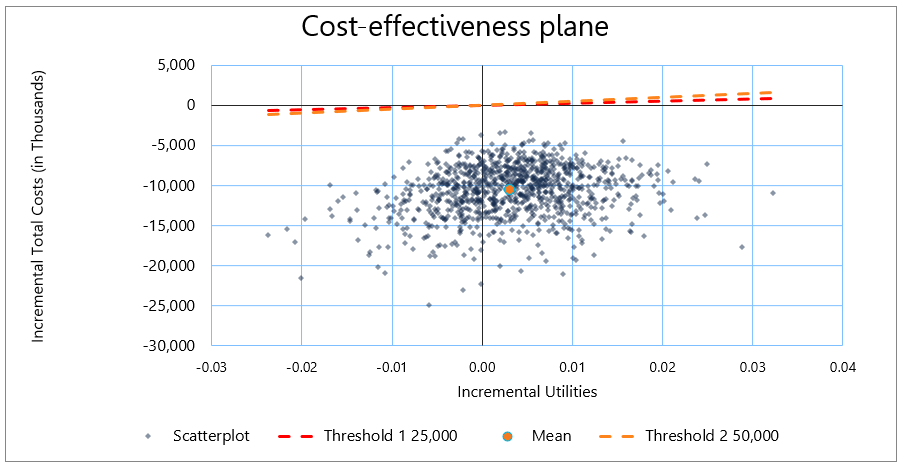 |
| --- |
