# Supplementary material for: Clinical and economic outcomes of adding [18F]FES PET/CT in estrogen receptor status identification in metastatic and recurrent breast cancer in the US
Source: PLoS One. 2024 May 14;19(5):e0302486. doi: 10.1371/journal.pone.0302486 (PMC11093585; doi:10.1371/journal.pone.0302486)
Supplement: S1 Table — (DOCX) [file pone.0302486.s010.docx]

| S1 Table. Results of the PSA (mean values)   \|  \| **[^18^F]FES PET/CT** \| **Biopsy/IHC** \| \| --- \| --- \| --- \| \| **Application (i) When biopsy failed or was inconclusive in mBC** \| \| \| \| Total cost (USD) \| 282,506 \| 283,570 \| \| Life years \| 2.389 \| 2.384 \| \| QALYs \| 1.277 \| 1.273 \| \| ICER \| Biopsy/IHC is dominated by [^18^F]FES PET/CT \| \| \| **Application (ii) When biopsy was not possible in mBC** \| \| \| \| Total cost (USD) \| 274,246 \| 284,919 \| \| Life years \| 2.404 \| 2.387 \| \| QALYs \| 1.284 \| 1.281 \| \| ICER \| Biopsy/IHC is dominated by [^18^F]FES PET/CT \| \| \| **Application (iii) When [^18^F]FES PET/CT in combination with IHC for rBC patients** \| \| \| \| Total cost (USD) \| 113,980 \| 121,457 \| \| Life years \| 1.434 \| 1.400 \| \| QALYs \| 0.452 \| 0.447 \| \| ICER \| Biopsy/IHC is dominated by [^18^F]FES PET/CT \| \|   [^18^F]FES PET/CT indicates 16α-[^18^F]fluoro-17β-fluoroestradiol with positron emission tomography imaging/computed tomography; ICER, incremental cost-effectiveness ratio; IHC, immunohistochemistry; mBC, metastatic breast cancer; PSA, probabilistic sensitivity analysis; QALYs, quality-adjusted life years; rBC, recurrent breast cancer. |
| --- | --- | --- | --- | --- | --- | --- | --- | --- | --- | --- | --- | --- | --- | --- | --- | --- | --- | --- | --- | --- | --- | --- | --- | --- | --- | --- | --- | --- | --- | --- | --- | --- | --- | --- | --- | --- | --- | --- | --- | --- | --- | --- | --- | --- | --- | --- | --- | --- |
